# Supplementary material for: Stable demographic ratios of haploid gametophyte to diploid sporophyte abundance in macroalgal populations
Source: PLoS One. 2024 Mar 7;19(3):e0295409. doi: 10.1371/journal.pone.0295409 (PMC10919683; doi:10.1371/journal.pone.0295409)
Supplement: S1 Appendix — (DOCX) [file pone.0295409.s002.docx]

**Appendix A: Density-independent model**

For tractability, first consider a species with an ideal isomorphic life cycle without asexual reproduction. Setting $\gamma=\gamma_{H}=\gamma_{D}$, $b=b_{H}=b_{D}$, $m=m_{H}=m_{D}$ yields the eigenvalues,

|  | $\lambda_{1}=1-m-\gamma b\sqrt{\sigma} \mathrm{and} \lambda_{2}=1-m+\gamma b\sqrt{\sigma}.$ | (A.1) |
| --- | --- | --- |

Hence, the leading right eigenvector for the leading eigenvalue $\lambda_{2}$ is $\left( \begin{matrix} 1/\sqrt{\sigma} \\ 1 \end{matrix} \right)$. This right leading vector gives the stable distribution of haploids in the population,

|  | $\rho_{H}=\frac{1}{1+\sqrt{\sigma}}.$ | (A.2) |
| --- | --- | --- |

This haploid frequency is equivalent to the classical result [1]. The result for an ideal isomorphic species without asexual reproduction suggests the more generalized frequency of haploids in the population (see details in the Supplementary *Mathematica* file).

**Appendix B: Fully sexual species with density dependence**

B1. Ideal isomorphic life cycle

Consider the case that $\gamma=\gamma_{H}=\gamma_{D}$, $b=b_{H}=b_{D}$, $m=m_{H}=m_{D}$, and $\alpha=\beta=1$; then the nontrivial equilibria are,

|  | $\hat{H}=-\frac{m+b\gamma\sqrt{\sigma}}{\delta m\left( 1-\sqrt{\sigma} \right)} \mathrm{and} \hat{D}=\frac{\left( m+b\gamma\sqrt{\sigma} \right)\sqrt{\sigma}}{\delta m\left( 1-\sqrt{\sigma} \right)},$ | (B.1a) |
| --- | --- | --- |
|  | $\hat{H}=\frac{b\gamma\sqrt{\sigma}-m}{\delta m\left( 1+\sqrt{\sigma} \right)} \mathrm{and} \hat{D}=\frac{\left( b\gamma\sqrt{\sigma}-m \right)\sqrt{\sigma}}{\delta m\left( 1+\sqrt{\sigma} \right)}.$ | (B.1b) |

Here Eq. (B.1b) is considered because the equilibrium point with a positive values should be biologically plausible (when $b\gamma\sqrt{\sigma}>m$). The haploid frequency at this equilibrium point gives us $\hat{H}/\left( \hat{H}+\hat{D} \right)=1/\left( 1+\sqrt{\sigma} \right)$, which is equivalent to Eq. (A.2).

The calculated eigenvalues of the stability matrix for the trivial equilibrium point (0, 0) are $-\gamma b\sqrt{\sigma}-m \mathrm{and} \gamma b\sqrt{\sigma}-m$. Hence, a haploid-diploid population exists when $\gamma b\sqrt{\sigma}>m$. Under this condition, the equilibrium described by Eq. (B.1b) exists in a positive region and is stable.

B2. General solution

Next, consider the case of a species in which the parameter values differ between haploids and diploids. In this case, strict solutions for the population densities at equilibrium are obtained. The biologically plausible equilibrium may be,

|  | $\hat{H}=\frac{\sqrt{w_{D}}\left( \sqrt{w_{H}w_{D}}-1 \right)}{\left( \sqrt{w_{H}}+\sqrt{w_{D}} \right)\delta} \mathrm{and} \hat{D}=\frac{\sqrt{w_{H}}\left( \sqrt{w_{H}w_{D}}-1 \right)}{\left( \sqrt{w_{H}}+\sqrt{w_{D}} \right)\delta},$ | (B.2) |
| --- | --- | --- |

where $w_{H}=\left( \sigma b_{H}\gamma_{D} \right)/{m_{D}}$ and $w_{D}=\left( b_{D}\gamma_{H} \right)/{m_{H}}$. Plugging these values into $T=\hat{H}+\hat{D}$ and $\rho_{H}=\hat{H}/\left( \hat{H}+\hat{D} \right)$, I obtain Eq. (6).

B3. Stability of a trivial equilibrium

The Jacobian matrix around the trivial equilibrium (0, 0) is,

|  | $\mathbf{J}_{0}=\left( \begin{matrix} -m_{H} & b_{D}\gamma_{H} \\ \sigma b_{H}\gamma_{D} & -m_{D} \end{matrix} \right).$ | (B.3) |
| --- | --- | --- |

Because the trace of this Jacobian matrix is always negative, the stability of the trivial equilibrium depends on the determinant of the matrix,

|  | $\det\left[ \mathbf{J}_{0} \right]=m_{H}m_{D}-\sigma b_{H}b_{D}\gamma_{H}\gamma_{D}.$ | (B.4) |
| --- | --- | --- |

The trivial equilibrium is stable when $\det\left[ \mathbf{J}_{0} \right]>0$. Therefore, it is unstable, and a haploid-diploid population can spread when rare if $\sigma b_{H}b_{D}\gamma_{H}\gamma_{D}>m_{H}m_{D}$ ($\det\left[ \mathbf{J}_{0} \right]<0$). This condition is equivalent to,

|  | $w_{H}w_{D}>1.$ | (B.5) |
| --- | --- | --- |

B4. Stability of a nontrivial equilibrium

Here, the stability of a nontrivial equilibrium, Eq. (B.2), is considered. The elements in the Jacobian matrix, $\mathbf{J}=\left( \begin{matrix} J_{11} & J_{12} \\ J_{21} & J_{22} \end{matrix} \right)$, around the equilibrium ($\hat{H},\hat{D}$) are,

|  | $J_{11}=\frac{b_{D}\gamma_{H}\left[ w_{H}^{2}+\sqrt{w_{H}w_{D}}+w_{H}\left( \sqrt{w_{H}w_{D}}-1-2w_{D} \right) \right]}{\left( w_{D}-w_{H} \right)\sqrt{w_{H}w_{D}}},$ | (B.6a) |
| --- | --- | --- |
|  | $J_{12}=\frac{b_{D}\gamma_{H}\left( 1+w_{D} \right)\left( 1-\frac{w_{H}}{\sqrt{w_{H}w_{D}}} \right)}{w_{D}-w_{H}},$ | (B.6b) |
|  | $J_{21}=\frac{\sigma b_{H}\gamma_{D}\left( 1+w_{H} \right)\left( \frac{\sqrt{w_{D}}}{\sqrt{w_{H}}}-1 \right)}{w_{D}-w_{H}},$ | (B.6c) |
|  | $J_{22}=-\frac{\sigma b_{H}\gamma_{D}\left[ w_{D}^{2}+\sqrt{w_{H}w_{D}}+w_{D}\left( \sqrt{w_{H}w_{D}}-1-2w_{H} \right) \right]}{\left( w_{D}-w_{H} \right)\sqrt{w_{H}w_{D}}},$ | (B.6d) |

The trace and determinant of this stability matrix are,

|  | $\mathrm{tr} \left[ \mathbf{J} \right]=\frac{b_{D}\gamma_{H}\sqrt{w_{H}}\left( 1-2\sqrt{w_{H}w_{D}}-w_{H} \right)+\sigma b_{H}\gamma_{D}\sqrt{w_{D}}\left( 1-2\sqrt{w_{H}w_{D}}-w_{D} \right)}{\sqrt{w_{H}w_{D}}\left( \sqrt{w_{H}}+\sqrt{w_{D}} \right)},$ | (B.7a) |
| --- | --- | --- |
|  | $\det\left[ \mathbf{J} \right]=\frac{2\sigma b_{H}b_{D}\gamma_{H}\gamma_{D}\left( \sqrt{w_{H}w_{D}}-1 \right)}{\sqrt{w_{H}w_{D}}}.$ | (B.7b) |

The trivial equilibrium is unstable (B.5) when $\det\left[ \mathbf{J} \right]>0$. Furthermore, because Eq. (B.5) indicates that $1<\sqrt{w_{H}w_{D}}<2\sqrt{w_{H}w_{D}}$, $\mathrm{tr} \left[ \mathbf{J} \right]<0$. Hence, when the trivial equilibrium is unstable, the nontrivial equilibrium, Eq. (B.2), is stable.

**Appendix C: Species with asexual reproduction**

C1. Stability analysis

The Jacobian matrix around the trivial equilibrium (0, 0) is,

|  | $\mathbf{J}_{0}=\left( \begin{matrix} -m_{H}+a_{H}b_{H}\gamma_{H} & \left( 1-a_{D} \right)b_{D}\gamma_{H} \\ \left( 1-a_{H} \right)\sigma b_{H}\gamma_{D} & -m_{D}+a_{D}b_{D}\gamma_{D} \end{matrix} \right).$ | (C.1) |
| --- | --- | --- |

The trace and determinant of this stability matrix are,

|  | $\mathrm{tr} \left[ \mathbf{J} \right]=-m_{H}-m_{D}+a_{H}b_{H}\gamma_{H}+a_{D}b_{D}\gamma_{D}=-m_{H}\left( 1-w_{H}^{A} \right)-m_{D}\left( 1-w_{D}^{A} \right),$ | (C.2a) |
| --- | --- | --- |
|  | $\det\left[ \mathbf{J} \right]=\left( m_{H}-a_{H}b_{H}\gamma_{H} \right)\left( m_{D}-a_{D}b_{D}\gamma_{D} \right)-\left( 1-a_{H} \right)\left( 1-a_{D} \right)\sigma b_{H}b_{D}\gamma_{H}\gamma_{D}$  $=m_{H}m_{D}\left[ \left( 1-w_{H}^{A} \right)\left( 1-w_{D}^{A} \right)-w_{H}^{S}w_{D}^{S} \right].$ | (C.2b) |

Hence, the trivial equilibrium is unstable and a haploid-diploid population can persist when $\mathrm{tr} \left[ \mathbf{J} \right]>0$ or $\det\left[ \mathbf{J} \right]<0$. If the fitness components of haploids and diploids are symmetrical for both the sexual and asexual reproduction, $w^{S}=w_{H}^{S}=w_{D}^{S}$ and $w^{A}=w_{H}^{A}=w_{D}^{A}$, then the trivial equilibrium is unstable when $w^{A}>1$ or $\left( w^{A}-1 \right)^{2}<\left( w^{S} \right)^{2}$. Hence, the sufficient condition for the existence of a haploid-diploid population is $w^{A}+w^{S}>1$. In contrast, the trivial equilibrium is stable when $w^{A}+w^{S}<1$.

Next, the stability of a nontrivial equilibrium is considered. Unfortunately, the trace and determinant of the stability matrix are difficult to show in simple form (Supplementary *Mathematica* file). When the fitness components of haploids and diploids for sexual and asexual reproduction are equal and the mortalities of haploids and diploids are also equal, then

|  | $\mathrm{tr} \left[ \mathbf{J} \right]=\frac{m_{H}+m_{D}}{2}\left( 1-w^{A}-3w^{S} \right),$ | (C.3a) |
| --- | --- | --- |
|  | $\det\left[ \mathbf{J} \right]=2m_{H}m_{D}w^{S}\left( w^{A}+w^{S}-1 \right).$ | (C.3b) |

Hence, the nontrivial equilibrium is stable when $w^{A}+w^{S}>1$. When the trivial equilibrium is unstable, it can be concluded that the nontrivial equilibrium is stable.

C2. Basic reproductive number

Consider the matrix $\mathbf{J}_{0}\boldsymbol{=}\mathbf{F-V}$ around the trivial equilibrium (Eq. (C.1)), where

|  | $\mathbf{F}=\left( \begin{matrix} a_{H}b_{H}\gamma_{H} & \left( 1-a_{D} \right)b_{D}\gamma_{H} \\ \left( 1-a_{H} \right)\sigma b_{H}\gamma_{D} & a_{D}b_{D}\gamma_{D} \end{matrix} \right)\mathrm{and}\mathbf{V}=\left( \begin{matrix} m_{H} & 0 \\ 0 & m_{D} \end{matrix} \right).$ | (C.4) |
| --- | --- | --- |

The next generation matrix (e.g., [2]), $\mathbf{F}\mathbf{V}^{-1}$, can be represented as,

|  | $\mathbf{M}=\left( \begin{matrix} \frac{a_{H}b_{H}\gamma_{H}}{m_{H}} & \frac{\left( 1-a_{D} \right)b_{D}\gamma_{H}}{m_{D}} \\ \frac{\left( 1-a_{H} \right)\sigma b_{H}\gamma_{D}}{m_{H}} & \frac{a_{D}b_{D}\gamma_{D}}{m_{D}} \end{matrix} \right)=\left( \begin{matrix} w_{H}^{A} & \frac{m_{H}}{m_{D}}w_{D}^{S} \\ \frac{m_{D}}{m_{H}}w_{H}^{S} & w_{D}^{A} \end{matrix} \right).$ | (C.5) |
| --- | --- | --- |

The largest eigenvalue of matrix $\mathbf{M}$ can be derived as,

|  | $R_{0}=\sqrt{\left( \frac{w_{D}^{A}-w_{H}^{A}}{2} \right)^{2}+w_{H}^{S}w_{D}^{S}}+\frac{w_{H}^{A}+w_{D}^{A}}{2}.$ | (C.6) |
| --- | --- | --- |

When asexual reproduction is ignored ($a_{H}=a_{D}$ and, thus, $w_{H}^{A}=w_{D}^{A}=0$), the basic reproductive number becomes $R_{0}=\sqrt{w_{H}^{S}w_{D}^{S}}=\sqrt{w_{H}w_{D}}$. Hence, the condition, $R_{0}>1$, is equivalent to Eq. (B.5).

**Appendix D: Different competition models**

In the main text, it is assumed that the density dependence of the population is regulated by mortality. Here, models with different modes of competition are shown to give a similar ploidy ratio (i.e., $\rho_{H}$ value).

D1. Strict density dependence

First, the case that the total population size is regulated to be constant by strict density dependence (i.e., held fixed at constant population size) is considered:

|  | $\frac{dH}{dt}=\left( m_{H}H\left( t \right)+m_{D}D\left( t \right) \right)\frac{\phi\left( t \right)}{\phi\left( t \right)+\psi\left( t \right)}-m_{H}H\left( t \right),$ | (D.1a) |
| --- | --- | --- |
|  | $\frac{dD}{dt}=\left( m_{H}H\left( t \right)+m_{D}D\left( t \right) \right)\frac{\psi\left( t \right)}{\phi\left( t \right)+\psi\left( t \right)}-m_{D}D\left( t \right).$ | (D.1b) |

Because Eqs. (D.1) assume that the total number of recruitments is equal to the total number of dead individuals, the population size is regulated to be constant (${dH}/{dt}+{dD}/{dt}=0$). Analysis of the model shows that the ploidy ratio at population equilibrium of Eqs. (D.1) is equal to that in Eq. (8b) (Supplementary *Mathematica* file).

D2. Logistic-type density dependence

Second, as in classical ecological and genetic models, a logistic-type density dependence with carrying capacity $K$ is assumed,

|  | $\frac{dH}{dt}=\gamma_{H}\phi\left( t \right)\left( K-H\left( t \right)-D\left( t \right) \right)-m_{H}H\left( t \right),$ | (D.2a) |
| --- | --- | --- |
|  | $\frac{dD}{dt}=\gamma_{D}\psi\left( t \right)\left( K-H\left( t \right)-D\left( t \right) \right)-m_{D}D\left( t \right).$ | (D.2b) |

Here, competition is assumed to regulate haploid and diploid abundance equally and carrying capacities of the different stages are assumed to be equal. Analysis of the model shows that the ploidy ratio at population equilibrium of Eqs. (D.2) is equal to Eq. (8b) (Supplementary *Mathematica* file).

D3. Density dependence via recruitment

To ensure a positive recruitment term, a model with competition for reproduction is:

|  | $\frac{dH}{dt}=\gamma_{H}\frac{\phi\left( t \right)}{1+\delta\left( H\left( t \right)+D\left( t \right) \right)}-m_{H}H\left( t \right),$ | (D.3a) |
| --- | --- | --- |
|  | $\frac{dD}{dt}=\gamma_{D}\frac{\psi\left( t \right)}{1+\delta\left( H\left( t \right)+D\left( t \right) \right)}-m_{D}D\left( t \right).$ | (D.3b) |

Here, competition is assumed to regulate haploids versus diploids equally and carrying capacities of different stages are equal, as in the model with density dependence via mortality. Analysis of the model shows that the ploidy at population equilibrium of Eqs. (D.3) is equal to Eq. (8b) (Supplementary *Mathematica* file).

**References**

[1] Thornber C. S., Gaines S. D. 2004. Population demographics in species with biphasic life cycles. Ecology 85: 1661-1674.

[2] Sato, K., 2019. Basic reproduction number of SEIRS model on regular lattice. Mathematical Biosciences and Engineering 16, 6708-6727.
