# Supplementary material for: Stable demographic ratios of haploid gametophyte to diploid sporophyte abundance in macroalgal populations
Source: PLoS One. 2024 Mar 7;19(3):e0295409. doi: 10.1371/journal.pone.0295409 (PMC10919683; doi:10.1371/journal.pone.0295409)
Supplement: Attachment — Submitted filename: Response_to_Reviewers.docx [file pone.0295409.s003.docx]

**Revisions (2023. 10. 11)**

**Reply to points by reviewer 1:**

[1-1]

*The author attempt to reconcile a long standing question about the maintenance of life cycles with more than one free-living stage using mathematical modeling. The stability of haploid and diploid stages in a population is the result of both sexual and asexual reproduction by each stage.*

*Overall there is very little integration of the results or synthesis with the literature in the discussion. This is a major weakness that needs to be addressed. Moreover, there is a lack of integration across the literature with what is known in algae with regard to life cycle and reproductive system from empirical data.*

I thank the reviewer for your helpful comments and have thoroughly revised the paper in response to your suggestions. Generally, I have majorly reorganized the Introduction and the Discussion. In particular, with the reviewers' comments in mind, the relevance and novelty of this study to previous studies about the evolution and ecology of life cycles are described more clearly.

In the following, we explain how we have revised the paper in response to each comment from the reviewer.

[1-2]

*L36: Not all algae can be considered plants. Thus, I would suggest to amend this first paragraph to focus on where haploid and diploid life cycles in such an alternation are found. In fact, they are found beyond 'plant' lineages in the Archaeplastids.*

Based on the reviewer’s suggestion, I have reorganized the head of the Introduction. This begins with the topic of more general eukaryotes rather than algae with regard to life cycle diversity.

[1-3]

*L39: Heteromorphic generations need not be marco vs. microscopic. Again, this paragraph is an over simplification of diversity.*

I agree. I have added the new section, “*Heteromorphic and isomorphic life cycles*” and explain explained the details of heteromorphic and isomorphic life cycles.

[1-4]

*L42: It is not a heteromorphic life cycle, but a heteromorphic alternation or heteromorphic generations.*

I know the term "heteromorphic (isomorphic) alternation of generations" is more classical. But recent research prefers the word "life cycle" (e.g., Thornber 2006, Coelho et al. 2007). With that in mind, I have defined both terms within a new section, "*Heteromorphic and isomorphic life cycles*".

[1-5]

*L43: Similarly, it is not an isomorphic life cycle.*

See reply of [1-4].

[1-6]

*L44: strange list of citations and unclear what you mean by tend to consist of haploid and diploid?*

Sorry for the confusion. I have reorganized the Introduction. I hope that the revised version would be better than the previous version (see the reply to [2-12]).

[1-7]

*L52-53: What does this mean?*

I mean that many studies are based on numerical calculations, and few studies derive simple analytical solutions. I have corrected the sentence.

[1-8]

*L60: Were there differences in Chondrus in biomechanical strength?*

In Carrington et al. (2001), authors reveal that gametophytic distal tissues were 43% stronger, 21% more extensible, and 21% stiffer than, tetrasporophytic distal tissues. These might be explained by following reason: Gametophyte with strong-gelling kappa-type carrageenan is mechanically superior to the sporophyte with nongelling lambda-type carrageenan. These details are not directly relevant to the context and were not discussed in the text.

[1-9]

*L63: Krueger-Hadfield and Ryan (2020) JPhycol showed differences in survivorship.*

Thank you for sharing the important information. I have cited this article (see reply to [2-21]).

[1-10]

*L69: Please see Stoeckel et al. 2021 J Hered for asexual reproduction. This paper is not referenced in the current manuscript but deals with sexual and asexual reproduction.*

Thank you for your comment. I have cited this article that pointed out the importance of asexuality. We note, however, that this article focuses on genetics but not on ecology (demography).

[1-11]

*L244: Switching between "i" and "we"*

Done.

[1-12]

*L261: Dependence?*

Thank you for pointing out a typo. I have corrected it.

[1-13]

*L276-277: No references? This claim needs support.*

I have cited the following article as the example of the asexual population, “Evolution and maintenance of haploid–diploid life cycles in natural populations: The case of the marine brown alga Ectocarpus (Couceiro et al. 2015, Evolution)”.

**Reply to points by reviewer 2:**

*I really enjoyed reading this submission. It is the kind of work that I personally like the most, both to do and to read. I am thankful to the author for giving me the pleasure of reading it.*

I thank the reviewer for his/her overall positive comments.

[2-1]

*The work is mathematically sound. Yet, for this work to be scientifically sound, it takes more than the mathematics. I have a few major criticisms that must be addressed:*

*The author must be clearer and more objective in his words, particularly concerning how the mathematics relate to (or model the) biology and ecology of these algae. Very important, there is a specific terminology (or nomenclature) that the author is neglecting (or misusing) and must be used (or corrected).*

*The work is poorly contextualized relative to these life-cycles and the most relevant theories and advances regarding their ecology and evolutionary stability.*

*This poor framing occurs when presenting the topic and the motive for doing this analysis, as well as when discussing the results and highlighting the key findings.*

*One criterion that is often used by other journals and publishers is the interest to the readers. I know from personal experience that, for the average audience in the field of algal ecology and evolution, deeply mathematical articles are not much appealing. This article is deeply mathematical and there is no way around it, nor do I wish so. Hence, for this article to be well taken by the respective scientific community, it must be very clarifying and convincing regarding the “so what?” factor. I suggest for the author to frame better this work regarding the several hypotheses advanced for the evolutionary stability of these life-cycles and unbalanced G:T ratios, and how this work advances the state-of-the art by supporting (or not) specific hypothesis.*

I thank the reviewer for your many helpful comments and have thoroughly revised the paper in response to your suggestions.

First, I have checked the accuracy of the wording in the manuscript and sought to make it accessible to readers who are not familiar with the biology of macroalgae, for example by explaining the details of their life cycles. We have also added the new chapter explaining the diversity in macroalgal life cycles and the diagram of these (Fig.1). Second, I have largely restructured the Introduction to make it easier to understand the relationship between this study and previous studies. Third, I have added a new section explaining how this study relates to and advances the ecological evolutionary research that has been accumulated to date.

*Below, I expand on these issues and present other minor issues. Although the list is large, all of them are rather easy to solve. Once they have been adequately addressed, I can only recommend the publication of this good work.*

Thank you for the positive comment! Below we address each of their suggestions.

[2-2]

*This work must have a figure and corresponding text detailing the isomorphic biphasic life-cycle. In fact, that was the standard in most of the literature here cited. This can be either part of the introduction or a special section after the introduction and prior to the model presentation. This presentation of the life-cycle must use the correct nomenclature, including keywords as gametophytes, tetrasporophytes, carposporophytes, meiosis, mitosis, syngamy, oogonia, spermatangia, gametangia, zygote, cystocarps, tetraspores, carpospores, etc.*

Thank you for the nice suggestion. I have added the new diagram (Fig.1) and chapter (“Life cycle of macroalgae”) describing the life cycle diversity in macroalgae. This new chapter is expected to help simplify the Introduction as well as help readers unfamiliar with the algal life cycle.

[2-3]

*This description must show that the life-cycle is actually triphasic (gametophytes, carposporophytes and tetrasporophytes) and clarify how it reduces to biphasic upon the assumption of male gametophytes fertilizing all female gametangia. It should also include the alternative haploid and diploid monophasic cycles set by sporogenesis (as in Hughes and Otto, 1999) that are fundamental in the present submission. Make a clear contrast between sporogenesis and gametogenesis.*

In the new section, “*Triphasic life cycle in red macroalgae*”, I have stated that most red algae (i.e., Florideophyceae) exhibit a triphasic life cycle and that this life cycle can be approximated as a biphasic life cycle alternating only between gametophyte and tetrasporophyte. In the new section, “*Alternation of nuclear phases and generations*”, I have explained that a monophasic life cycle (diploidy) is observed in some species (e.g., Fucales).

[2-4]

*Here and elsewhere along the manuscript, the author must clarify that his “asexual reproduction” is only sporogenesis, and that he is disregarding vegetative growth (in red algae, the growth of new fronds from the same holdfast or from broken fragments).*

In the new section, “*Asexual reproduction in macroalgae*”, I have clarified that asexual reproduction includes vegetative growth and that we modeled only sporogenesis in the article.

[2-5]

*Here and elsewhere along the manuscript, the author must be aware of the difference between fertility and fecundity. Broadly speaking, fecundity is the production of newborns. But these must survive to be recruited into the population. In this case, this comprehends spore survival while suspended in the water column, settling in suited substrate, germination and survival as germlings until juvenile thalli. Fertility is the final outcome of fecundity and spore survival.*

Thank you for the important comment. I have corrected the terminology as you suggest. I define the *b* as the “fecundity”.

[2-6]

*The author should use this presentation of the life-cycle to expand on the main questions and theories about its ecology and evolution, and to frame his work relative to these questions and theories. The article by Hughes and Otto (1999) is a very good guide to follow.*

Based on the reviewer’s suggestion, I have added the section explaining the previous theoretical research about the evolution of the life cycle (especially the evolution of haploid versus diploid).

[2-7]

*I tend to like focused, succinct text. However, this introduction is too short. It provides a vague, incomplete presentation of the topic. Sometimes it takes steps too large while overlooking issue that are important to better understand the presentation.*

I have reorganized the Introduction completely with your suggestions. I hope that the revised version would be better than the previous version.

[2-8]

*The author must also be clearer about haploids and diploids being phenotypically similar. Such statement (in several parts of the text) is vague. Is the author referring only to the thalli or also the spores and germlings? Regarding morphology, eco-physiology and bio-mechanics, which affect survival and growth, or also regarding fecundity and spore survival?*

*I dislike the idea of ecologically similar corresponding to phenotypically similar because, in my perspective, phenotype also comprehends the reproductive biology, which can never be similar among gametophytes and tetrasporophytes. Although it is perfectly understandable what the author is trying to say, I would never use such wording and I do not recall having ever red it elsewhere.*

I have reorganized the Introduction and added the new section, “*Heteromorphic and isomorphic life cycles*”. In the new text, I have tried to be as accurate as possible (see also reply [2-14]).

[2-9]

*The author never presents the case that the dominance patterns (differences in abundances) follow a spatial or seasonal pattern, and that this indicates ecological and niche differentiation between haploids and diploids.*

I could understand the importance of the dominance patterns following a spatial or seasonal pattern. However, mentioning spatio-temporal variation would be confusing to the reader. After carefully considering this point, I have decided to add the sentence “The dominance patterns of two stages following spatial or seasonal patterns are often observed in the field, however for tractability, constant life history parameters and a well-mixed population are assumed.” in the model section. We note that the importance of the seasonal oscillation is touched in the “Future perspectives”.

[2-10]

*Lines 36-37: Algae are not plants.*

I have reorganized the head of the Introduction. The Introduction begins with the topic of more general eukaryotes rather than algae with regard to life cycle diversity (see [1-2]).

[2-11]

*Lines 41-44: This is a poor explanation of the specificities of heteromorphic and isomorphic biphasic life-cycles. I would merge with the previous sentences. I would first write all need be said about the heteromorphic case and only afterwards pass to the isomorphic case. In my opinion, the “going back and forth” along lines 38 to 44 is not ideal.*

I have reorganized the Introduction. I have added the new section, “*Heteromorphic and isomorphic life cycles*”. I hope that the revised version would be better than the previous version.

[2-12]

*The sentence “In contrast, populations of species with an isomorphic life cycle tend to consist of both haploid and diploid individuals” is particularly unfortunate because both heteromorphic and isomorphic biphasic life-cycles consist of both haploid and diploid free-living individuals. The stressor here is that, in isomorphic biphasic life-cycles, haploids and diploids are apparently morphologically similar, and thus expected to also be ecologically similar and occupy the same niche. However, experimental studies have proved that gametophytes and tetrasporophytes are not exactly morphologically similar, much less eco-physiologically or bio-mechanically.*

I agree that this sentence is confusing. I have reorganized the sentence.

As the reviewer suggests, the strict definition of the classification of heteromorphy versus isomorphy is difficult. However, compared to extremely heteromorphic life cycle species, the two stages of isomorphic species clearly occupy ecologically close niches (e.g., Ulva, Hiraoka and Yoshida 2010, Journal of Phycology), suggesting that some sort of disruptive selection may be at work, but this issue is probably still open. After carefully considering the advice of the reviewer, I decided to address this issue in a new section (*Heteromorphic and isomorphic life cycles*), and I chose not to go into it in depth in the Introduction.

[2-13]

*Line 46: “… field studies on [not of] the ratio of haploid-to-diploid abundances [plural] have shown both the occurrence of haploid-dominated (refs) and diploid-dominated (refs) populations”. It would also be nice to present how this ratio has been named (H:D or G:T).*

Thank you for your comment. I have added the sentence describing these ratios.

[2-14]

*Line 49-50 “haploid versus diploid dominance [not abundance] patterns”. The issue is the dominance.*

Thank you for your comment. I have corrected “abundance” into “dominance”.

[2-15]

*Line 55: “haploids and diploids have identical phenotypes” is misleading. It was gametophytes and tetrasporophytes (i.e., the adult thalli) but not the tetraspores and carpospores (i.e., the spores, which are also either haploid or diploid). And they were compared under the assumption of identical survival and growth rates. I believe that phenotype also comprehends the reproductive biology of the respective individuals. The reproductive biology of gametophytes and sporophytes is inevitably widely different without prejudice of morphological similarity and niche overlap.*

This is important comment. I have corrected this sentence as “Thornber and Gaines (2004) presented an analytical solution only for the case where haploids (gametophytes) and diploids (tetorasporophytes) have identical survival and growth rates”.

[2-16]

*The abundance ratio (actually proposed by Destombe et al 1989 and Scrosati et al 1999, before Thornber and Gaines 2004) is a theoretical hypothesis grounded on the ecological similarity between gametophytes and tetrasporophytes and the differences between their reproductive biologies. This is not the same as the assumption that they are phenotypically similar. In fact, they cannot be phenotypically similar given that they have different reproductive structures and biologies. This goes on throughout the text (line 57 and henceforth). My comment applies to all these instances.*

I have reorganized the main text. Especially, I have avoided the wording, “identical phenotype” (see [2-8], [2-12], and [2-15]).

[2-17]

*Lines 59-64: All 6 lines within the same brackets is not a good way to write. Brackets should be for small intermissions. The text should be reformulated to be generally outside brackets. Krueger-Hadfield and Ryan (2020) also found differences in survival rates.*

Based on the reviewer’s suggestion, I have put the text outside of the bracket.

[2-18]

*Vieira et al. (2021) also found differences in growth rates. Usandizaga et al. (2023) also found differences in resistance to epiphyte infection. Thornber et al. (2006) and Verges et al. (2008) also found differences in resistance to herbivory. Many other examples exist.*

Thank you for sharing the important information. I have cited these articles. Because the macroalgae in Verges (2008) exhibit a heteromorphic life cycle, however, I skipped it. Thornber et al. (2006) has been cited already.

[2-19]

*Lines 65-71: “To address the shortcomings in theoretical treatments of the demography of macroalgal populations” is very vague. The author did not even debate properly the theoretical hypothesis about - and modelling of - the ecology and evolution of isomorphic biphasic life cycles, much less their shortcomings.*

Thank you for the comment. I have reorganized the Introduction. There, I have explained the previous studies so that the novelty of this article can be understood.

[2-20]

*Lines 69-70: This is not quite true. Vieira and Santos (2010) addressed the effect of vegetative growth of new fronds from the same holdfast (it is included in what they call the looping rates). Here, and throughout the manuscript, the author only refers to sporogenesis. However, asexual reproduction also comprehends vegetative growth, fragmentation, budding, binary fission, etc.*

In the Introduction, I have cited the previous articles that consider the asexual reproductions (see [2-19]).

[2-21]

*Lines 71-72: This is Discussion and not Introduction.*

Following the comment of reviewer, I have reorganized the sentence.

[2-22]

*Line 77: I would not brand the matrix model by Thornber and Gaines (2004) as “the classical”. Other authors have also developed matrix models of isomorphic biphasic life-cycles (ex: Engel et al, 2001; Vieira and Santos, 2010, 2012a, 2012b; Vieira and Mateus, 2014) which are not less relevant. It should be enough rephrasing to “updating the matrix model by Thornber and Gaines (2004)”.*

Based on the reviewer’s suggestion, I remove the term “classical”.

[2-23]

*Lines 75-93: The model presentation is mathematically correct … provided that the incorrect use of nomenclature is corrected.*

Thank you for the important comment. I have corrected the terminology as you suggest.

[2-24]

*“haploid and diploid reproductive cells” are actually the haploid and diploid spores, namely the tetraspores and carpospores.*

The model does not assume red algae (Florideophyceae) only. The term "reproductive cell" is used to enhance the generality of the model. After considering the reviewer's comment, I have added the terms "tetraspores and carpospores" with parentheses.

[2-25]

*“in flowing water” is while suspended in the water column.*

*“the survivorship of haploid and diploid reproductive cells in flowing water is ...,” is actually the survival of tetraspores and carpospores while suspended in the water column.*

I have corrected the text as “while suspended in the water column”.

[2-26]

*“the fertility of the haploid and diploid stages per individual is ...” is actually the fecundity.*

*Fertility is bgamma.*

I have corrected the term “fertility” into “fecundity”.

[2-27]

*Adult survival is 1-m.*

I have added “(adult survival is 1 - *m*)” in the main text.

[2-28]

*“Fractions aH and aD of the reproductive outputs of the haploid and diploid stages are asexual, skipping the sexual loop and developing directly into haploids and diploids.” Aside the poor English, this is sporogenesis.*

I have added the term, “sporopgenesis”.

[2-29]

*Line 78: This is a poor way to start presenting the basics of the model. I propose. “the haploid (H) and diploid (D) densities are forecasted to time t+1 from their realized densities at time t and their vital rates (equation (1)”*

I have corrected the sentence as the reviewer suggests.

[2-30]

*The Discussion does an interesting presentation of the importance of sexual and asexual reproduction, and of the new metric developed, the Reproductive number. However, it does a poor job framing these new findings in the context of the theories and questions about the ecology and evolution of this life-cycle. In fact, there are only as little as 3 citations in the Discussion, one of them being a self-citation. Several interesting questions and works are overlooked:*

*- the relative importance of fertility, growth and survival rates, and of differences between haploids and diploids in these vital rates (see works by Hughes and Otto, Fierst, Thornber and Gaines, Vieira, among others).*

*- the conditions for fixation in biphasic vs monophasic life-cycles (see works by Hughes and Otto, 1999; Hal, 2000; Bessho and Otto, 2022, among others).*

*- The cost of sex (see Richerd et al, 1993)*

*- The cost of DNA (see Lewis, 1985; Mable 2001)*

In the Discussion, I have added a new section, “*Ecology and evolution of haploid-diploid life cycles*”, explaining how this study relates to and advances the previous ecological evolutionary research and how this study contributes to future empirical researches. We note that the "ecological problems" are important to treat the "evolutionary problems" but these two problems are not similar. Discussion of these two problems simultaneously would have the risk of confusing readers, I think. After considering, I don't cite the article about the nutrient-limitation hypothesis (Lewis, 1985) in the Discussion.

**Journal Requirements:**

[3-1]

*1. Please ensure that your manuscript meets PLOS ONE's style requirements, including those for file naming.*

Done.

[3-2]

*Please remove any funding-related text from the manuscript and let us know how you would like to update your Funding Statement.*

Done

[3-3]

*Please state what role the funders took in the study. If the funders had no role, please state: "The funders had no role in study design, data collection and analysis, decision to publish, or preparation of the manuscript." Please include this amended Role of Funder statement in your cover letter; we will change the online submission form on your behalf.*

Done.

[3-4]

*4. In your Data Availability statement, you have not specified where the minimal data set underlying the results described in your manuscript can be found. PLOS defines a study's minimal data set as the underlying data used to reach the conclusions drawn in the manuscript and any additional data required to replicate the reported study findings in their entirety. All PLOS journals require that the minimal data set be made fully available. For more information about our data policy, please see http://journals.plos.org/plosone/s/data-availability.*

Since this is a theoretical study, results are asserted by mathematical proof, not data. We note, however, that the Mathematica code used for the proof is available for download as the Supplementary file.

Thank you for all the useful comments by the reviewers. I hope that the revised version would be clearer than the previous version.
