# Supplementary material for: Stable demographic ratios of haploid gametophyte to diploid sporophyte abundance in macroalgal populations
Source: PLoS One. 2024 Mar 7;19(3):e0295409. doi: 10.1371/journal.pone.0295409 (PMC10919683; doi:10.1371/journal.pone.0295409)
Supplement: Attachment — Submitted filename: Response_to_Referees.docx [file pone.0295409.s004.docx]

**Revisions (2023. 11. 21)**

**Reply to points by reviewer 1:**

[1-1]

*Overall this manuscript is substantially improved and all my original comments have been addressed. However, a minor detail is to carefully go through the manuscript and choose to either use "we" or "I" as the manuscript goes back and forth between the two.*

Thank you for your positive comment. I have double-checked and change some “we” into “I”.

**Reply to points by reviewer 2:**

*The author addressed all my comments and suggestions in a very satisfactory way. In some places, the integration of his results and analytical solutions with former literature was very well achieved (see lines 262-270 for a good example). The outcome is good and I recommend its publication. There are, nevertheless, some issues still to be addressed/corrected, from conceptualization to grammar. I detail below.*

Thank you for your recommendation. Below I address revises for your suggestions.

[2-1]

*This work focuses on the effects of ploidy differences in fitness (W). The analytical solutions derived and the figures presented have W as basis. However, for W contribute sexual and asexual reproduction, but also survival (here, mortality m) (see W derivations in the text), but its effects were overlooked. Aside, growth rates were entirely neglected, and these can also be important.*

*Previous works showed that ploidy differences in growth and survival rates can be of utmost importance. However, here, ploidy differences in m were overlooked and the work reads as if it all depended exclusively from ploidy differences in sexual and asexual reproduction (as example, see Discusion\ lines 362-366). This is false. The author would arrive at the same results if the ploidy differences in W were driven by ploidy differences in m. However, throughout the work the author always considers that mH and mD are equal.*

*Grounding my claims, a few lines below the author states “this study explicitly considers density dependence, which many previous studies have omitted for simplicity. Many previous studies (e.g., Vieira and Santos 2010; 2012) define the ploidy ratio by the right eigenvector in the matrix model, where the density dependence is ignored and the population size diverges to infinity. In reality, the population size should remain finite and the stable ploidy ratio of this population is important.”*

*Therefore, in the author’s own words, the factors constraining population growth and causing density-dependence are fundamental. Hence, ploidy differences in these factors are also fundamental for the ploidy ratio as well as the evolutionary stability of isomorphic biphasic life cycles. Well, the factors behind density-dependency are growth and survival, which the author presently overlooked. Even in Appendix D, if I understood well, the different modes of density-dependency are never tested considering the possibility of ploidy differences in survival/mortality.*

*I take this chance to clarify that the effects of density-dependency (both Allee effects (detrimental low densities) and self-thinning (detrimental high densities)), and its ploidy differences, where evaluated by Vieira et al (2022) applying an Individual Based Model to Gracilaria chilensis (aka Agarophyton chilense).*

As a result of my analysis, the various life history parameters include of both mortality rates and birth rates are contracted as the fitness components. Hence, the parameter dependence of the fitness components has full information of the parameter dependence of all life history parameters. I would appreciate if the review could agree that I never assume equal mortality rates in the analysis. In particular, since growth rate may be positively correlated with birth rate directly, differences in growth rates between haploids and diploids would be able to be interpreted as differences in fitness. On the other hand, I agree that the parameter dependence of mortality rates can be a bit confusing. As I describe in answer [2-2], the fitness component is defined as the fecundity of the target individual divided by the mortality rate of different individuals. To avoid this confusion, I have added the parameter dependence of mortality rates by new paragraphs.

About the density dependence, in Equations (2), I assume that the mortality rates increase with the biomass. As reviewer pointed out, the density dependence is assumed to be equal between haploids and diploids. If we consider the difference in the parameters $\delta$ between haploids and diploids, we can test the effect of difference in ploidy in mortality rates. Unfortunately, this effect complicates the analysis and we no longer get a simple result. In the section “*Future Perspective*”, I have added the importance of the difference in the density dependence.

For the exitance of the previous study considering density dependence, I have cited this pioneering study in the Introduction.

[2-2]

*Another proof, and simultaneously a consequence, of overlooking survival are the sentences in lines 282-285: “The ploidy ratio and biomass both depend on haploid fitness () and diploid fitness (). Because haploids reproduce diploid individuals and vice versa, haploids are dominant in a population with higher diploid fitness.” This is misleading, with two aspects contribute to it:*

*1) This is only true in species where their demography (and their fitness) is dominated by fertility rates, or eventually by growth rates with fecundity being proportional to frond size. However, the exact opposite happens in species for which their demography (and fitness) is dominated by survival and/or clonal growth. The current biased analysis and conclusion resulted from the author not having tested for the effects of different degrees of m and of ploidy differences in m. Fierst et al. (2005) demonstrated that the patterns of ploidy dominance are highly dependent on the amount of m. Vieira and Santos (2010), as well as proceeding works by Vieira, corroborated it and advanced by showing that for species that mainly invest in survival (i.e., low m), ploidy differences in survival (in m) or clonal growth are the dominant factor determining patterns of ploidy dominance.*

*2) The traditional concept of fitness is the ability of producing offspring (i.e., next generation). This is straight-forward in mono-phasic life cycles. However, in biphasic life cycles, the offspring (i.e., the next generation) is of the opposite ploidy. Hence, the fitness components comprehend the vital rates necessary to get to the opposite ploidy phase, and not to complete the full life cycle. Once this is clarified, the fitness components of the sexual loop, as presented in the text, become clear. And lines 282-285 make more sense. But if this is not clarified ahead, the reader may become confused with the W of the sexual loop and lines 282-285.*

*I see 2 alternatives: 1) The author clarifies that here he focuses in the reproductive aspects and in due time the author will produce further work addressing the effects of growth and survival rates. I understand that addressing them all in just one article is unfeasible. Hence, this should be the first article in a series dedicated to this study. I myself have done the same for the same reasons.*

*2) In the proper places throughout the text, the author clarifies that m is also a fundamental component of W. Simultaneously, the author corrects the sentences that misleadingly suggest that W is only (or primarily) determined by reproduction. The later goes in accordance with the author argumentation in the discussion “Of course, the measurement of individual life history parameters remains important, but in the future, when classifying and understanding the diversity of ploidy ratios observed in the field, the question of how the three components are balanced is more essential than the question of which life history traits are important”. I can accept this. Just make clear throughout the text that survival/mortality is also a fundamental aspect of the fitness components (w).*

Thank you for important comment. I agree. My definition of fitness components could be confusing because, fitness is usually defined as the fecundity of the target individual divided by the mortality rate, whereas the fitness component is defined as the fecundity of the target individual divided by the mortality rate of different individuals ($w_{H}=\left( \sigma b_{H}\gamma_{D} \right)/{m_{D}}$ and $w_{D}=\left( b_{D}\gamma_{H} \right)/{m_{H}}$). To avoid this confusion, I have explicitly stated that the mortality rates in the components are not that of the target individual when I define the fitness components. In addition, the parameter dependence of mortality rates has been added in the main text.

[2-3]

*Sometimes the text is too wordy and redundant.*

I have revised the main text again.

[2-4]

*Lines 37-43: “The life cycle of sexual eukaryotes is characterized by the alternation of two nuclear phases: haploid and diploid. This alternation of nuclear phases results in an alternation of generations between two ploidy stages (haploid and diploid stages) and a biphasic life cycle is observed in many plants, algae, and fungi (Raper and Flexer 1970; Willson 1981; Valero et al. 1992; Klinger 1993; Bell 1997; Mable and Otto 1998; Coelho et al. 2007). In particular, in many macroalgae, the free-living haploid gametophytes and free-living diploid sporophytes alternate (details are described in the next section).”*

*This first paragraph of the introduction is vague explaining the main concept but still too wordy in some sentences. The concept of biphasic life-cycle is only presented in the last sentence. However, it is debated since the first sentence. These can lead the less-experienced reader to confusion. I give an example of how it may be improved:*

*Sexual reproduction in eukaryotes leads to the alternation of two nuclear phases, namely the haploid (n) and diploid (2n). In many plants, algae, and fungi both ploidy stages develop into adult free-living organisms and as such, a biphasic life cycle is observed (Raper and Flexer 1970; Willson 1981; Valero et al. 1992; Klinger 1993; Bell 1997; Mable and Otto 1998; Coelho et al. 2007). Many macroalgae alternate between a free-living haploid generation (the gametophytes) and the free-living diploid generation (the sporophytes).*

Following the review’s comment, I have reorganized the sentences.

[2-5]

*Line 44-45: It is redundant to say “… this alternation of generations (biphasic life cycle) …”. It was already well explained above.*

Following the review’s comment, I have shorted the paragraph.

[2-6]

*Line 48-50: This sentence is confusing. Does the author intend to say that there may be species with heteromorphic biphasic life-cycles, yet with minor phenotypic differences? Maybe the author means: “Species with the heteromorphic life cycle show large phenotypic differences (e.g., macroscopic versus microscopic, erect versus crustose). These species also often show different seasonal appearances and habitats (Higa et al. 2007).”*

Following the review’s comment, I have shorted the paragraph.

[2-7]

*Line 51: “with” instead of “exhibiting the”.*

Done.

[2-8]

*Line 55: “interesting” and not “interested”.*

Done.

[2-9]

*Line 55-57: This sentence is confusing because it does not clarify that it is haploid and diploid life cycles that are being mentioned, and not haploid and diploid individuals or generations, as mentioned earlier. Also, I do not see how this sentence leads to its following sentence. The text in lines 57-61 need a better introduction to it. Overall, this paragraph needs a better introductory explanation.*

Following the review’s comment, I have reorganized the paragraph. In particular, the order was rearranged so that the paragraph of previous studies on the evolution of diploidy was introduced first, followed by the studies on algal life cycles.

[2-10]

*Lines 69-71: “… have shown that both …”. Also, it should be clarified that patterns of haploid or diploid dominance have been observed over different taxa, different populations of the same species, and even over the same population depending on the special location or season.*

Following the review’s comment, I have added the sentences, “different patterns of haploid or diploid dominance have been observed for different populations of the same species, and even over the same population depending on the special location or season (De Wreede and Green 1990; Mudge and Scrosati 2003; Thornber and Gaines 2003; Dyck and De Wreede 2006)”.

[2-11]

*Line 94: “analyses”.*

Thank you for your check. I have corrected this typo.

[2-12]

*Line 95-97: “… and aims to express stable ploidy ratios in a haploid-diploid population using a mathematical expression as simple as possible.”*

I have corrected this sentence.

[2-13]

*Lines 101-104: The author should explicitly mention that hereafter “asexual reproduction” means sporogenesis. As it is, it suggests that sporogenesis is the only form of asexual reproduction, which is incorrect.*

Following the review’s comment, I have corrected the sentence about the Bessho and Otto (2022).

[2-14]

*Line 105: “… analyse the right eigenvector …” is jargon too specific. Maybe “… analyse the population structure yield by a matrix model …” is more accessible to the common reader.*

Following the review’s comment, I have corrected the sentence about the Vieira and Santos (2010; 2012).

[2-15]

*Line 107: Please, do not start a paragraph with “Furthermore …”.*

I have started from “in addition” and combined two paragraphs.

[2-16]

*Line 133: “the brown algae Fucales, …”. Otherwise, with the “brown algae, Fucales, …” reads as if all brown algae are Fucales and have a diploid life cycle.*

I have corrected the sentence as “individuals belonging to Fucales, a group of brown algae, are diploid”.

[2-17]

*Lines 137-139: “Since this article focuses on the dynamics of both gametophytes and sporophytes, I assume that the alternation of generation between haploid gametophyte and diploid sporophyte always occurs.” Besides unnecessary, this sentence is misleading and untruthful. Throughout the article, and as soon as in the Abstract, the author claims:*

*“Furthermore, the persistence of a haploid-diploid population and its total biomass are shown to be determined by the basic reproductive number (0), which is shown to be a function of these four demographic forces. When R0 is greater than one, the haploid- diploid population stably persists, and the ploidy ratio obtained by the analytical solution is realized”*

*This implies that the haploid-diploid population may not persist, with one of the phases being eliminated, namely when R0<1. This contradicts the claim above that “… I assume that the alternation of generation between haploid gametophyte and diploid sporophyte always occurs.”. No, you don’t. And you even determine the conditions for one of them to be eliminated. The conditions for such evolutionary non-stability of isomorphic biphasic life cycles have been studied before.*

I agree. I have deleted this sentence.

[2-18]

*Lines 152-157: “I note that, however, even when there are substantial differences between gametophytes and sporophytes, they may not be extreme. For example, the species of brown algae, Ectocarpus, both gametophytes and sporophytes are small and filamentous, but there is a substantial morphological difference between them. This species is classified as “heteromorphic (near-isomorphic)” (Couceiro et al. 2015).”*

*I do not see the point of these text.*

*#1 – first sentence only says that “substantial” differs from “extreme”. Dictionaries already do that.*

*#2 – second sentence only says that heteromorphicity may encompass other features besides size and filamentous. That happens with other species, even of red algae, as is Asparogopsis armata and Falkenbergia rufolanosa, which are actually the haploid and diploid phases of the same life cycle.*

*Better English: “In the brown algae Ectocarpus, both …”*

I have corrected the sentences as “I note that the difference between gametophytes and sporophytes is sometimes less extreme. In the brown algae Ectocarpus, for example, both gametophytes and sporophytes are small and filamentous, but there is a substantial morphological difference between them. This species is classified as heteromorphic (near-isomorphic)”.

[2-19]

*Lines 157-160: poor English. Change to “The accumulation of empirical studies showing that gametophyte and sporophyte (or tetrasporphyte of red algae) phenotypes differ (morphologically, ecophysiologically, or biomechanically) calls into question the classical classification of the life cycle into heteromorphic and isomorphic categories.”*

Following the review’s comment, I have corrected the sentences.

[2-20]

*Lines 165-16: poor English. Change to “Among red algae, however, many species from the Florideophyceae are known to exhibit more complex life cycles. Here, the female gametes fertilized on the haploid female gametophyte develop into diploid multicellular structures, the carposporophytes …”. Furthermore, this is a vague description. More detail would be preferable.*

Following the review’s comment, I have corrected the sentences as “Here, syngamy occurs when a haploid sperm without flagella (spermatium) attaches to haploid female gametes (carpogonium) that attaches on the female gametophyte. This fertilized cell develops into diploid multicellular structure, the carposporophyte”.

[2-21]

*Line 168: First, the carposporophyte produces diploid carpospores by somatic cell division. Only later they are released.*

Following the review’s comment, I have corrected the sentences as ”First, the carposporophyte produces diploid carpospores by somatic cell division. Only later they are released and these develop into free-living diploid sporophytes, tetrasporophytes”.

[2-22]

*Line 171-172: “three phases” and not “three alternating generations” as the carposporophyte is not free-living and thus not a generation. By calling the carposporophyte a generation, the author is contradicting himself in his former presentation of the subject (see above). “Triphasic” means 3 phases, not 3 generations.*

I have corrected the word “generations” into “phases”.

[2-23]

*Line 174-175: Just to make sure, I went again through the article by Kamiya and Kawai (2002), specifically dedicated to the carposporophytes. Nowhere do they call it an “independent phase”, much less “generation”. We agree that this article is the reference about carposporophytes. So, lets agree that the carposporophyte is not an “independent phase”, much less a “generation”.*

Following the review’s comment, I have corrected the sentences as “Because the ploidy of carposporphyte (diploid) is different from gametophyte (haploid), the caposporophyte is recognized as a distinguishable phase, but is nutritionally dependent (parasitic) on the female gametophyte”.

[2-24]

*Lines 203-212: The explanation of the rationale for bH/2 is vague and could be improved. The bH is the fecundity rate of haploid females (i.e., production of carposporophytes). However, not all haploids are females as some are males. Assuming a 1:1 sex ratio, ½ of the haploids are females. Hence, in the matrix model, haploid fecundity is bH/2. Then, comes the reproductive cost f, leading to fbH/2. In my opinion, this explanation is clearer than the current text.*

Following the reviewer’s comment, I have reorganized the sentences.

[2-25]

*Line 336: I believe there is o typo as W_H^S is repeated twice and there is no W_D^S.*

Thank you for pointing out the mistake! I have corrected it.

[2-26]

*Lines 378-383: citing those studies might be adequate.*

Following the review’s comment, I have sited the previous paper.

[2-27]

*Line 382: “… an increase in its …”.*

I have corrected it.

[2-28]

*Line 388: Is this true for the work by Hughes and Otto (1999)?*

I have cited Hughes and Otto (1999).

[2-29]

*Line 405: “when the sexual reproduction term is smaller and can be ignored”. This would be better placed in line 403, before the equation: “Interestingly, the model presented here suggests that, when the sexual reproduction term is smaller and can be ignored, the basic reproductive number can be approximated as”*

Done.

[2-30]

*Line 418-419: This English is grammatically incorrect. There is number disagreement (singular and plural mixed) and it I am not sure what the author intends to say. The sentence must be revised. Maybe “… in haploid-diploid life-cycles …”?*

I have deleted “a” from the sentence.

[2-31]

*Line 427: The Individual Based Model by Vieira et al. (2022) is the more recent and most complex of them.*

I have cited Vieira et al. (2022).

**Journal Requirements:**

[3-1]

*Please review your reference list to ensure that it is complete and correct. If you have cited papers that have been retracted, please include the rationale for doing so in the manuscript text, or remove these references and replace them with relevant current references. Any changes to the reference list should be mentioned in the rebuttal letter that accompanies your revised manuscript. If you need to cite a retracted article, indicate the article’s retracted status in the*

I have re-checked the citation list again.

Thank you for all the useful comments by the reviewers. I hope that the revised version would be clearer than the previous version.
